# *ETV6::ABL1*-Positive Myeloid Neoplasm: A Case of a Durable Response to Imatinib Mesylate without Additional or Previous Treatment

**DOI:** 10.3390/ijms25010118

**Published:** 2023-12-21

**Authors:** Maria Teresa Bochicchio, Giovanni Marconi, Carmen Baldazzi, Lorenza Bandini, Francesca Ruggieri, Alessandro Lucchesi, Claudio Agostinelli, Elena Sabattini, Agnese Orsatti, Anna Ferrari, Giorgia Capirossi, Chiara Servili, Andrea Ghelli Luserna di Rorà, Giovanni Martinelli, Giorgia Simonetti, Gianantonio Rosti

**Affiliations:** 1 Biosciences Laboratory, IRCCS Istituto Scientifico Romagnolo per lo Studio e la Cura dei Tumori (IRST) “Dino Amadori”, 47014 Meldola, FC, Italy; francesca.ruggieri@irst.emr.it (F.R.); anna.ferrari@irst.emr.it (A.F.); giorgia.capirossi@studio.unibo.it (G.C.); chiara.servili@irst.emr.it (C.S.); giorgia.simonetti@irst.emr.it (G.S.); 2 Hematology Unit, IRCCS Istituto Scientifico Romagnolo per lo Studio e la Cura dei Tumori (IRST) “Dino Amadori”, 47014 Meldola, FC, Italy; giovanni.marconi@irst.emr.it (G.M.); alessandro.lucchesi@irst.emr.it (A.L.); 3 Istituto di Ematologia “Seràgnoli”, IRCCS Azienda Ospedaliero-Universitaria di Bologna, 40138 Bologna, BO, Italy; carmen.baldazzi@gmail.com (C.B.); lorenza.bandini.92@gmail.com (L.B.); 4 Department of Medical and Surgical Sciences (DIMEC), University of Bologna, 40100 Bologna, BO, Italy; claudio.agostinelli@unibo.it; 5 Haematopathology Unit, IRCCS Azienda Ospedaliero-Universitaria di Bologna, 40138 Bologna, BO, Italy; elena.sabattini@aosp.bo.it (E.S.); agnese.orsatti@studio.unibo.it (A.O.); 6 Fondazione Pisana per la Scienza ONLUS, 56017 San Giuliano Terme, PI, Italy; a.ghelli@fpscience.it; 7 Scientific Directorate, IRCCS Istituto Scientifico Romagnolo per lo Studio e la Cura dei Tumori (IRST) “Dino Amadori”, 47014 Meldola, FC, Italy; giovanni.martinelli@irst.emr.it

**Keywords:** myeloid/lymphoid neoplasm, *ETV6::ABL1*, fusion genes, imatinib mesylate, next generation sequencing, diagnostic RNA panels

## Abstract

*ETV6::ABL1* rearranged neoplasms are rare hematological diseases. To date, about 80 cases have been reported, including myeloid and lymphoid leukemias. The *ETV6* gene codes for an ETS family transcription factor and several fusion partners have been described. When translocated, *ETV6* causes the constitutive activation of the partner genes. Here, we report the case of a 54-year-old woman with a cryptic insertion of the 3′ region of *ABL1* in the *ETV6* gene. The patient was first diagnosed with idiopathic hypereosinophilic syndrome, according to the clinical history, conventional cytogenetics, standard molecular analyses and pathologist description. Next generation sequencing of diagnosis samples unexpectedly detected both *ETV6::ABL1* type A and B fusion transcripts, which were then confirmed by FISH. The diagnosis was Myeloid/Lymphoid neoplasm with *ETV6::ABL1* fusion, and the patient received imatinib mesylate treatment. In a follow-up after more than one year, the patient still maintained the molecular and complete hematological responses. This case highlights the importance of timely and proper diagnostics and prompt tyrosine kinase inhibitor treatment.

## 1. Introduction

*ETV6::ABL1* (also known as *TEL*::*ABL1*) is a rare fusion that has been found in different types of hematological diseases. To date, about 80 cases of hematological neoplasms (more frequently, acute lymphoblastic leukemia (ALL), myeloproliferative neoplasms (MPNs), Philadelphia-negative chronic myeloid leukemia (CML) [1], atypical (a)CML and chronic myelomonocytic leukemia (CMML)) carrying *ETV6::ABL1* translocation have been reported [2,3,4,5,6]. ETV6::ABL1 chimeric protein has been found in less than 1% of ALL cases; however, due to the lack of a systematic screening for this fusion transcript, its overall incidence in hematological neoplasms cannot be precisely defined [2]. The *ETV6* (ETS variant 6) gene encodes for an ETS family transcription factor containing two functional domains: the N-terminus PNT (exons 3–4) and the C-terminus ETS (exons 6–7) domains, flanking the central domain coded by the exon 5 [7]. ETV6 is involved in the maintenance of the vascular network, hematogenesis, embryogenesis and development of different tissues [7]. The *ABL1* gene encodes for a non-receptor tyrosine kinase containing different structural domains, including SRC-homology domains (SH1, SH2 and SH3) responsible for the regulation of their own activity, DNA-binding (DB) domains and actin-binding (AB) domains, in addition to a nuclear translocation signal (NTS) sequence, sites for phosphorylation by protein kinase C (PKC) and a proline-rich sequence [8]. Two types of *ETV6::ABL1* in-frame fusion isoforms have been described: the so-called “type A”, involving exon 4 of *ETV6* and exon 2 of *ABL1*, and the “type B” translocation, involving exon 5 of *ETV6* and exon 2 of *ABL1*. The ETV6::ABL1 fusion protein retains both the SH domains and the tyrosine kinase domain of ABL1 [9], leading to the loss of ABL1 autoinhibitory activity, thus resulting in a constitutive active enzyme. Both transcripts encode for a chimeric non-receptor tyrosine kinase resembling the *BCR*::*ABL1* structure. Moreover, in vitro studies have demonstrated that ETV6::ABL1 phosphorylates the same substrates activated by BCR::ABL1 chimeric proteins, suggesting that ETV6 may replace the BCR role and activate ABL1 [10], and explaining why patients harboring *ETV6::ABL1* translocation are sensitive to TKI treatment [9]. Here, we present the case of a patient who received a diagnosis of Myeloid/Lymphoid neoplasm with *ETV6::ABL1* fusion and reached a durable response by imatinib mesylate treatment.

## 2. Case Description

**Figure 1:**
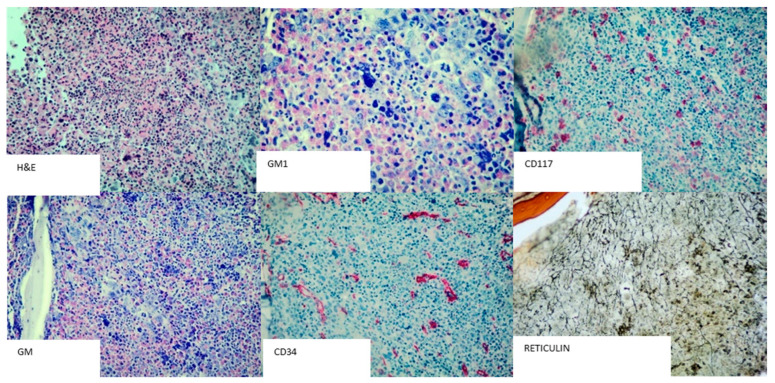
Morphological characterization. Hematoxylin and eosin (H&E, 200×) and Giemsa stain (GM, magnification 200×; GM1, magnification 400×); immunohistochemistry (IHC) stains for CD34 and CD117 (magnification 200×) and Gomori stain for reticulin fibers (Reticulin, magnification 200×).

**Figure 2:**
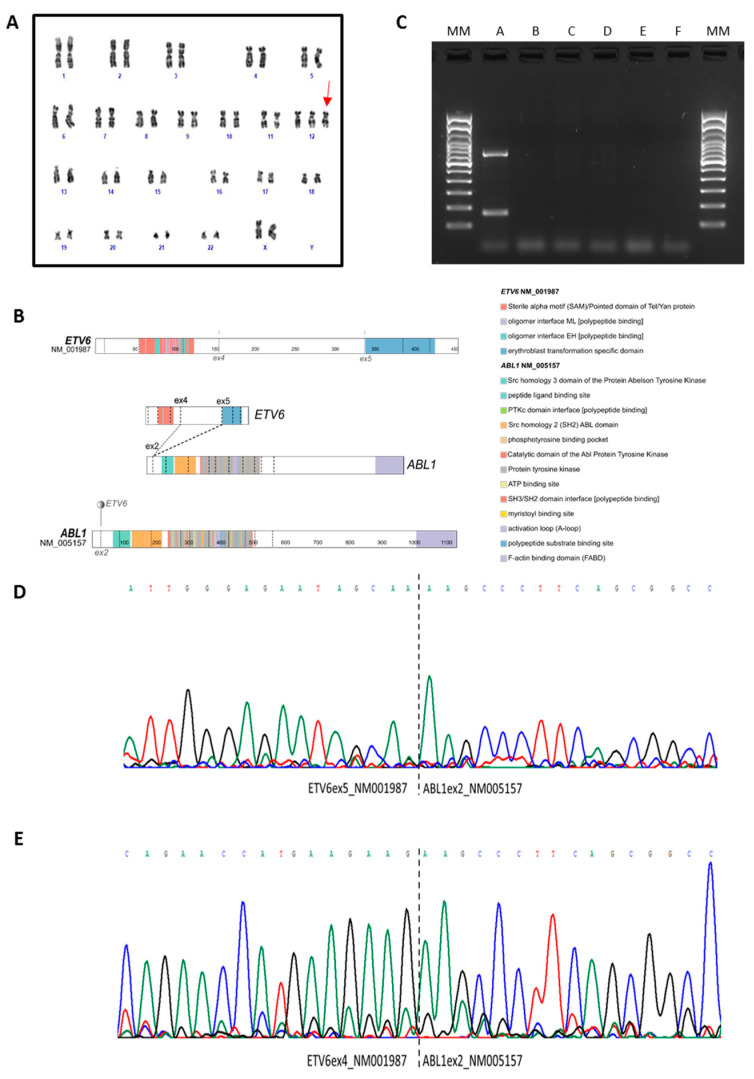
Cytogenetic and molecular characterization. (**A**) G-banded karyotype with trisomy involving the chromosome 12 (red arrow). (**B**) Overview of the *ETV6::ABL1* fusion transcripts detected by NGS and involving exon 5 of *ETV6* and exon 2 of *ABL1*, or exon 4 of *ETV6* and exon 2 of *ABL1*, respectively. (**C**) RT-PCR revealing the 706 bp *ETV6exon5::ABL1exon2* fusion transcript (upper band) and the 160 bp *ETV6exon4::ABL1exon2* fusion transcript (lower band). A 100 bp molecular weight marker was used. (**D**,**E**) Electropherograms of the sequences spanning the breakpoint confirming in-frame fusions involving *ETV6* exon 5 or *ETV6* exon 4 and *ABL1* exon 2. The dashed lines indicate the breakpoint regions. Each peak represents a single nucleotide in the DNA sequence, and each nucleotide has a different colour; A is green, T is red, C is blue and G is black. MM: Molecular Marker; A: diagnosis; B: 3 months follow-up; C: 6 months follow-up; D: 12 months follow-up; E: RT negative; F: PCR negative.

In January 2019, a 54-year-old Caucasian woman was referred to our institution for leukocytosis. She had a mild increase in white blood cell (WBC) count over one year (mean WBC 12 × 10^9^/L, mean neutrophils 8 × 10^9^/L), basophilia (5%) and eosinophilia (14%). *JAK2*, *CALR* and *MPL* mutations and *BCR*::*ABL1* rearrangements were negative (peripheral blood). The patient was asymptomatic and was not receiving any chronic treatment. She had no significant medical history, no history of smoking, no ongoing infections, negative inflammation markers, a normal chest and abdomen examination, and a normal abdomen ultrasonography (US). The patient underwent a regular follow-up (every 4 months) and did not receive any treatment. In the following 18 months, the WBC counts fluctuated around 11–13 × 10^9^/L and the clinical patients’ conditions were stable. In June 2020, the WBC count was raised to 32 × 10^9^/L (basophilia 4%, eosinophilia 11%), while hemoglobin and platelets count were within the normal range, spleen was not palpable and the abdomen US results were normal. *BCR*-*ABL1* translocation and *JAK2*, *CALR*, and *MPL* mutations were confirmed to be negative (peripheral blood). Furthermore, no *PDGFRA*, *PDGFRB* or *FGFR1* rearrangements were detected. A trephine biopsy showed hypercellular bone marrow (95%) with a diffuse eosinophilic infiltration, slightly reduced erythropoiesis, normal CD34^+^ cells and mastocytes (Figure 1). Cytogenetic examination showed a unique clone characterized by 47, XX, +12 (on 20 metaphases, Figure 2A). Chest X-ray and heart US excluded any significant organ involvement. Therefore, the patient was disagosed with idiopathic hypereosinophilic syndrome.

Next generation sequencing (NGS) was performed on the diagnosis peripheral blood sample, on both DNA and RNA, using the Oncomine Myeloid Assay (Thermo Fisher Scientific, Waltham, MA, USA), surprisingly revealing the presence of both *ETV6*::*ABL1* type A and B fusion transcripts (Figure 2B and Table S1), while confirming the absence of the Philadelphia chromosome. The *ETV6*::*ABL1* fusions were confirmed by RT-PCR (Figure 2C) and Sanger sequencing (Figure 2D,E). No additional DNA variants were found. Further information on the sample collection, nucleic acids isolation, library preparation and RT-PCR are reported in the Supplementary Methods. The variants and fusions tested are reported in Tables S2–S4.

**Figure 3:**
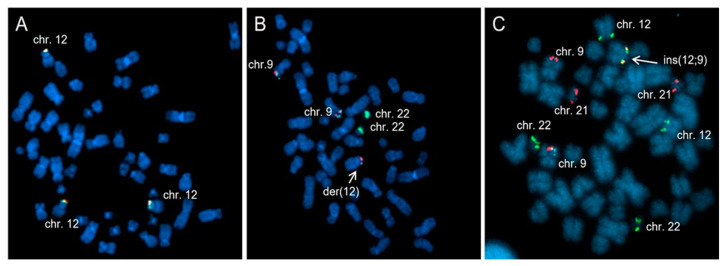
FISH analyses (100× magnification). (**A**) FISH analysis with *ETV6* break-apart on previously G-banded metaphase showing 3 fusion signals on 3 chromosomes 12, indicating 3 copies of *ETV6*. (**B**) FISH analysis with *BCR*-*ABL1* TCDF probes on previously G-banded metaphase showing 2 green signals on chromosome 22, two blue/red signals on chromosome 9 and an extra red signal on the short arm of chromosome 12, indicating *ABL1* rearrangement. (**C**) FISH analysis combining *ETV6-RUNX1* DCDF and *BCR-ABL1* TCDF probes on metaphase showing a fusion between *ETV6* marked in spectrum green and *ABL1* marked in spectrum orange on derivative chromosome 12, confirming *ETV6::ABL1* rearrangement. The arrows indicate the derivatives chromosome.

In order to confirm the fusion transcripts revealed by NGS, we performed fluorescent in situ hybridization (FISH). FISH analysis using an *ETV6* break-apart probe revealed three copies of the *ETV6* gene without evidence of *ETV6* rearrangement (Figure 3A). On the contrary, FISH analysis with a *BCR::ABL1* Tricolor Color Dual Fusion (TCDF) probe confirmed the presence of the *ABL1* rearrangement with *ABL* located on one chromosome 12 (Figure 3B). In order to confirm the *ETV6*::*ABL1* fusion, we performed FISH analysis combining *ETV6::RUNX1* ES Dual Color Dual Fusion and *BCR::ABL1* TCDF. FISH analysis showed the presence of the *ETV6*::*ABL1* fusion on chromosome 12 (Figure 3C). Therefore, we concluded that the *ETV6*::*ABL1* fusion was the result of a cryptic insertion of the 3′ of *ABL1* (q34) into the *ETV6* locus (12p13). Details on chromosome banding analysis (CBA) and FISH are described in the Supplementary Methods. Based on the results, the diagnosis was modified to Myeloid/Lymphoid neoplasm with *ETV6::ABL1* fusion, and the patient started imatinib mesylate treatment at the dose of 200 mg QD (8 September 2020).

**Figure 4:**
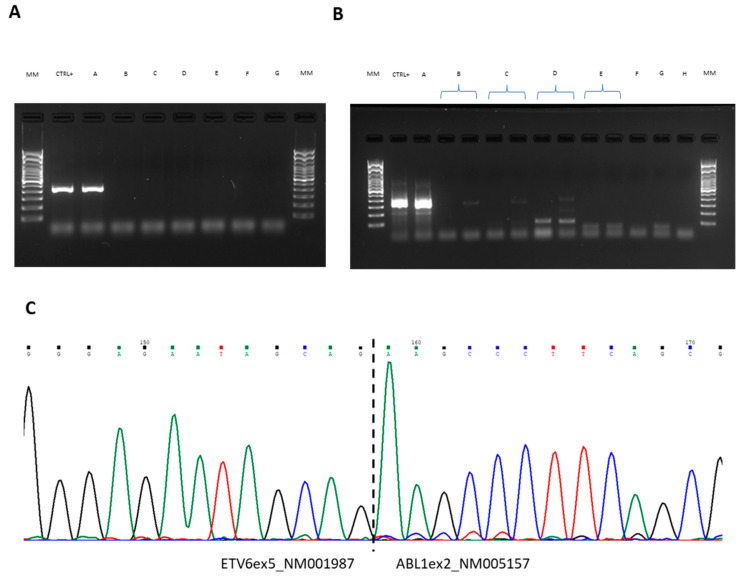
Monitoring of *ETV6*exon5::*ABL1*exon2 fusion overtime on peripheral blood samples. (**A**) RT-PCR revealing the 421-bp *ETV6*exon5-*ABL1*exon2 fusion transcript. (**B**) Nested PCR revealing the 321-bp *ETV6*exon5::*ABL1*exon2 fusion transcript. A 100 bp molecular weight marker was used. (**C**) Electropherogram of the sequence spanning the breakpoint that confirmed in-frame fusion involving *ETV6* exon 5 and *ABL1* exon 2. The dashed lines indicate the breakpoint regions. Each peak represents a single nucleotide in the DNA sequence, and each nucleotide has a different colour; A is green, T is red, C is blue and G is black. MM: Molecular Marker; A: diagnosis; B: 3 months follow-up; C: 6 months follow-up; D: 12 months follow-up; E: 18 months follow-up; F: RT negative; G: PCR negative; H: Nested-PCR negative.

**Figure 5:**
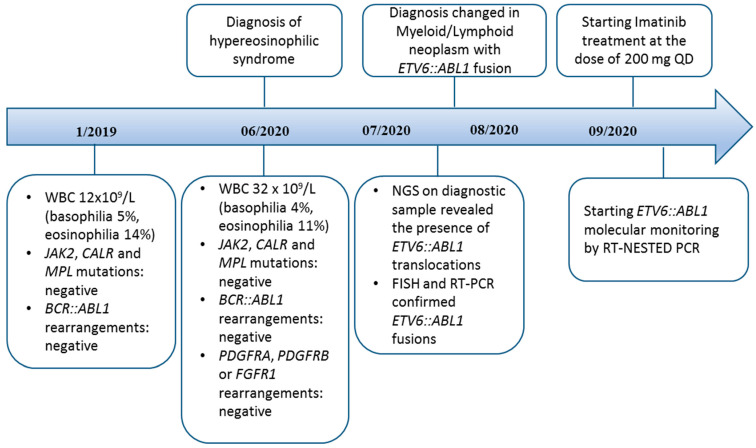
Timeline displaying clinical and therapy schedule (upper part) and laboratory data (bottom part).

We monitored the most abundant fusion transcript, *ETV6*exon5::*ABL1* exon2, using both RT-PCR and Nested PCR. Follow-up peripheral blood samples were collected and analyzed at 3 months (time point B), at 6 months (time point C), at 12 months (time point D) and at 18 months (time point E) of therapy, according to clinical practice. RT-PCR results were negative in all the follow-up samples (Figure 4A). Negativity was confirmed by NGS at time points B and C using the Myeloid Plus Solution panel (Sophia Genetics). Nested-PCR negativity was reached at time point D, while samples at time points B and C were still weakly positive (1/2 replicates, Figure 4B). The complete hematological response was assessed by blood count, revealing all the parameters to be within the normal range. The last evaluation—after 3 years of imatinib mesylate treatment—showed a WBC count of around 5 × 10^9^/L and a neutrophils count of 3.70 × 10^9^/L (basophilia 0.6%, eosinophilia 2.4%). The main clinical and laboratory information are summarized in Figure 5.

## 3. Discussion

In this study, we report the case of an *ETV6::ABL1* rearranged patient with a diagnosis of Myeloid/Lymphoid neoplasm, who received imatinib mesylate treatment and achieved a durable response.

The formation of an in-frame *ETV6::ABL1* fusion gene involves complex genomic rearrangements because *ETV6* and *ABL1* genes have opposite chromosome orientations. Conventional diagnostic techniques (such as conventional cytogenetics) sometimes fail to detect this rearrangement because of its cryptic nature due to the similar G-banding pattern of the distal long arm of chromosome 9 and the distal short arm of chromosome 12. Moreover, no ready-to-use *ETV6::ABL1* FISH probes are commercially available, suggesting that the *ETV6-ABL1* fusion may remain undetected in a number of patients [5,11]. 

In the case we present, the use of next generation sequencing allowed us to overcome the above-mentioned limitations and to detect this fusion transcript, which changed the initial diagnosis of hypereosinophilic syndrome into the proper diagnosis of Myeloid/Lymphoid neoplasm with *ETV6::ABL1* fusion.

The patient showed the presence of both type “A” and “B” fusion transcripts, first detected by NGS and then confirmed by RT-PCR and Sanger sequencing. FISH analysis combining *ETV6::RUNX1* DCDF and BCR-ABL1 TCDF probes performed on metaphases showed a cryptic insertion of the 3′ region of *ABL1* in the *ETV6* gene on one chromosome 12, in addition to a signal consistent with trisomy 12.

*ETV6*::*ABL1* rearrangements have been reported in different hematological malignancies and, in particular, in ALL, followed by MPNs and acute myeloid leukemia (AML) [11,12]. Eosinophilia represents a common clinical feature and a hallmark of all *ETV6::ABL1* rearranged MPNs cases reported in the literature [2], while the most frequent molecular alterations observed in ALL or in lymphoid blast crisis (LCB) patients are deletions of *CDKN2A/CDKN2B*, *IKZF1* or *PAX5* [2].

*ETV6*::*ABL1* rearranged hematological neoplasms share many clinical features with CML. Indeed, the ETV6::ABL1 fusion protein functionally resembles the BCR::ABL1 ones, being characterized by a constitutive activation of the chimeric transcript [13], and sensitivity to both first- [14] and second-generation tyrosine kinase inhibitor (TKI) treatments [15]. For these reasons, the patient received imatinib therapy. Imatinib competitively binds the ABL1 ATP binding site in the ETV6::ABL1 fusion protein by the same mechanism of action described for the BCR::ABL1 protein.

Despite this, in the literature, few patients have received TKI treatment at first manifestation of the disease and/or at the first progression, and most of them died or relapsed/evolved [2]. Schwaab et al. presented data on *ETV6*::*ABL1* rearranged MPN patients that received imatinib, nilotinib or dasatinib after a prior treatment with hydroxyurea and/or cytarabine or intensive chemotherapy. Patients receiving imatinib did not achieve a complete cytogenetic (CCR) or molecular (CMR) response, which was instead obtained by patients under nilotinib or dasatinib treatment [16]. Accordingly, previous reports showed that imatinib allowed only an initial reduction of disease followed by a mild neutrophilia, basophilia and eosinophilia after 6 months, with persistent *ETV6*::*ABL1* positivity in FISH and nested PCR [13], or a transient response followed by transformation into ALL [17]. Moreover, most patients reported in the literature were diagnosed as atypical AML or Ph-like ALL.

Conversely, our patient presented without any blast excess at the diagnosis, and the clinical features were consistent with chronic diseases. She started TKI treatment as a frontline therapy after receiving the correct diagnosis and is still maintaining complete hematological and molecular responses after 36 months of imatinib mesylate. To our knowledge, this is the first case reporting a follow-up longer than one year without any additional [13] or previous chemotherapy treatment [18]. Our data likely rule out the co-occurrence of genomic events accounting for resistance in our patient and suggest a potential driver role for the *ETV6::ABL1* rearrangement. Studies accounting for different sensitivity profiles, gene expressions and BCR-ABL1-like signatures are warranted in this case; however, an accurate and multicenter sample collection is needed to meet this endpoint.

## 4. Conclusions

The detection of the *ETV6::ABL1* rearrangement remains difficult, due to its cryptic nature.

A deeper genomic characterization of patients with *ETV6::ABL1* fusion transcripts may improve our understanding of the biological complexity behind this disease. Although this could represent a limitation of our study, overall, our results underline the importance of timely and proper diagnostics, and the need to revise the current screening algorithms—for example, by recommending NGS RNA panels—in order to detect and monitor *ETV6::ABL1* rearrangements. In this case, molecular biology was instrumental in the diagnosis and, consequently, in the selection of an appropriate therapy. Notably, in the absence of molecular biology results, supportive therapies would have been the only ones administered to the patient.

## Data Availability

All data generated or analyzed during this study are included in this published article and in its Supplementary Materials.

## Supplementary Materials

The following supporting information can be downloaded at: https://www.mdpi.com/article/10.3390/ijms25010118/s1. References [19,20,21] are cited in the supplementary materials.
