# Supplementary material for: ETV6::ABL1-Positive Myeloid Neoplasm: A Case of a Durable Response to Imatinib Mesylate without Additional or Previous Treatment
Source: Int J Mol Sci. 2023 Dec 21;25(1):118. doi: 10.3390/ijms25010118 (PMC10779409; doi:10.3390/ijms25010118)
Supplement: Supplementary file 1 [file ijms-25-00118-s001.zip › ijms-2713049-supplementary.pdf]

Supplementary Materials

Methods

Sample collection

The study was approved by Comitato Etico della Romagna (protocol 5805/2019, #NCT04298892) and was carried out in accordance with the principles laid down in the 1964 Declaration of Helsinki. Written informed consent was received from the patient prior to inclusion in the study, along with written consent to publish the information contained in this article. Samples were collected in EDTA tubes, for sequencing analysis, and Heparin tubes for cytogenetics analysis, at diagnosis, at +3, +6, +12 and +18 months of treatment according to the clinical practice.

DNA and RNA isolation

Genomic DNA and total RNA were isolated from white blood cells using Maxwell® RSC Blood DNA Kit and simplyRNA Blood Kit (Promega Corporation, Madison, USA) respectively, and have been quantified using Qubit 4.0 (Thermo Fisher Scientific, Waltham, Massachusetts, USA).

Trephine bone marrow biopsy (BMB) and Immunohistochemistry (IHC)

The BMB was fixed in 10% buffered formalin, decalcified in ethylenediaminetetraacetic acid tetrasodium salt (EDTA)-based solution, and paraffin embedded. Hematoxylin and Eosin and Giemsa stains were analyzed. IHC was performed as previously reported [19], using the following antibodies: anti-CD34 (clone QBEnd/10), anti-CD117 (clone EP10).

Next Generation Sequencing

The mutational status of 30-40 genes together with the principal translocations involved in myeloid malignancies were studied using Oncomine™ Myeloid Research Assay (OMRA, Thermo Fisher Scientific, Waltham, Massachusetts, USA) and/or Myeloid Plus solution (SOPHiA Genetics, Sulpice, Switzerland) at diagnosis and during follow up respectively, according to the manufacturer's instructions. The OMRA library were prepared using the Ion Chef System (Thermo Fisher Scientific, Waltham, Massachusetts, USA) according to the manufacturer's instructions. A library was prepared for DNA and RNA simultaneously on the same chip for each sample and loaded onto an Ion 530 chip (Thermo Fisher Scientific, Waltham, Massachusetts, USA) using Ion 510 & Ion 520 & Ion 530 Kit-Chef. Sequencing was performed on an Ion S5 sequencer (Thermo Fisher Scientific, Waltham, Massachusetts, USA). GrCh37 (hg19) was used to align sequences and base calling was performed using Torrent Suite (V.5.10). Alignment reads were estimated using Ion Reporter (IR) software (V.5.10) providing absolute read counts and read counts per million (RPM) [20]. Myeloid Plus Solution libraries were prepared according manufacturer's instructions. The median average of DNA amplicons was determined by capillary electrophoresis using Agilent High Sensitivity DNA kit (Agilent Technologies, Inc., Santa Clara, California, USA). DNA and RNA libraries were pooled together with the following ratio 97%/3% and were paired-end (2×301) sequenced with v3 chemistry on a MiSeq™ instrument (Illumina, Inc., San Diego, California, USA), as described in the manufacturer's protocol. FASTQ sequencing files were analyzed by SOPHiA DDM® platform. Human Genome Build 19 (Hg19) was used as the reference for sequence alignment. The fusion transcripts found in diagnostic samples by Oncomine Myeloid Reaserch Assay are listed in Supplementary Table 1. Supplementary Tables 2-4 list the covered genes and translocations assessed by NGS, respectively.

Chromosome Banding Analysis (CBA) and Fluorescence in Situ Hybridization (FISH)

CBA was performed on bone marrow cells as previously reported [21]. FISH analysis was carried out on fixed nuclei and previously GAW-banded metaphases obtained from CBA technique, according to manufacturer's recommendations. FISH was performed with LSI ETV6 Breakapart probe (Vysis, Abbott Molecular, Illinois, USA), LSI ETV6 (TEL)-RUNX1 (AML1) ES Dual Color Translocation Probe Set and BCR/ABL1/ASS1 Tri-Color DF FISH Probe (Vysis, Abbott Molecular, Illinois, USA). The slides were counterstained with DAPI and analyzed using fluorescent-microscopes equipped with FITC/TRITC/AQUA/DAPI filter sets and the Genikon imaging system software (Nikon Instruments, Tokyo, Japan). At least 200 nuclei were analyzed.

RT-PCR and Nested-RT-PCR

One hundred ng of RNA has been reverse-transcribed using SuperScript VILO cDNA Synthesis Kit (Thermo Fisher Scientific, Waltham, Massachusetts, USA) as follows: 25°C for 10 minutes, 42°C for 60 minutes, 85 °C for 5 minutes followed by a hold at 4°C. In order to confirm the presence of both ETV6exon4-ABL1exon2 and ETV6exon5-ABL1exon2 fusion transcripts the following program was followed: 95°C for 5 minutes; 95°C for 30 seconds, 60.8°C for 30 seconds and 72°C for 30 second (35 amplification cycles); 72°C for 7 minutes and a final hold at 4°C. To specifically identify the *ETV6exon5-ABL1exon2*, 20 ng of cDNA was used to perform polymerase chain reaction (PCR) with the FastStart High Fidelity PCR System and a ready-to-use solution of PCR grade nucleotides (Roche) as follow: 95°C for 5 minutes; 95°C for 30 seconds, 65°C for 30 seconds and 72°C for 30 second (35 amplification cycles); 72°C for 7 minutes and a final hold at 4°C. For nested-PCR the previously amplification thermal profile was used. Primers used for PCR and nested-PCR are listed below. PCR amplicons were purified using QIAquick PCR Purification Kit (QIAGEN) according to manufacturer's instructions. Samples were sequenced according to dideoxy procedure BigDye® Terminator v3.1 Cycle Sequencing Kit (Thermo Fisher Scientific) on a 3130 Series Genetic Analyzer (Thermo Fisher Scientific Inc, Applied Biosystem™, Foster City, CA 94404, USA) and sequences were analyzed using GeneMapper™ Software 5 (Thermo Fisher Scientific). All the sample were evaluated for actin expression using the following amplification program: 95°C for 5 minutes; 95°C for 30 seconds, 68°C for 30 seconds and 72°C for 30 second (35 amplification cycles); 72°C for 7 minutes and a final hold at 4°C.

The following primers were used for PCR and nested-PCR:

**Primer**	**Sequence (5′→3′)**	**Reference/website**
Fw *ETV6*exon4	TCTATACACACACAGCCGGA	Primer3web, version 4.1.0
Rev *ABL1*exon2	TGGGGTCATTTTCACTGGGT	Primer3web, version 4.1.0
Fw *ETV6*exon5 (TEL-A)	TGCACCCTCTGATCCTGAAC	(doi: 10.1038/sj.leu.2401592).
Rev *ABL1*exon2 (ABL1-a3-B)	GTTTGGGCTTCACACCATTCC	(doi: 10.1038/sj.leu.2401592).
Fw *ETV6*exon5- (TEL-C) (nested-PCR)	AAGCCCATCAACCTCTCTCATC	(doi: 10.1038/sj.leu.2401592).
Rev *ABL1*exon2 (ABL-a3-D) (nested-PCR)	TTCCCCATTGTGATTATAGCCTA	(doi: 10.1038/sj.leu.2401592).
Fw *Actin*	TTGTTACAGGAAGTCCCTTGCC	(doi:10.1016/j.canlet.2016.12.006.)
Rev *Actin*	ATGCTATCACCTCCCCTGTGTG	(doi:10.1016/j.canlet.2016.12.006.)

References

19. Righi, S.; Novero, D.; Godio, L.; Bertuzzi, C.; Bacci, F.; Agostinelli, C.; Sagramoso, C.; Rossi, M.; Piccioli, M.; Gazzola, A.; et al. Myeloid Nuclear Differentiation Antigen: An Aid in Differentiating Lymphoplasmacytic Lymphoma and Splenic Marginal Zone Lymphoma in Bone Marrow Biopsies at Presentation. Hum. Pathol. 2022, doi:10.1016/j.humpath.2022.03.008.
20. Jeon, M.J.; Yu, E.S.; Kim, D.S.; Choi, C.W.; Kim, H.N.; Ah Kwon, J.; Yoon, S.Y.; Yoon, J. Performance Evaluation and Clinical Impact of the Oncomine Myeloid Research Assay for Gene Expression Analysis in Myeloid Haematologic Malignancies. J. Clin. Pathol. 2022, doi:10.1136/jcp-2022-208425.
21. Simonetti, G.; Padella, A.; do Valle, I.F.; Fontana, M.C.; Fonzi, E.; Bruno, S.; Baldazzi, C.; Guadagnuolo, V.; Manfrini, M.; Ferrari, A.; et al. Aneuploid Acute Myeloid Leukemia Exhibits a Signature of Genomic Alterations in the Cell Cycle and Protein Degradation Machinery. Cancer 2019, doi:10.1002/cncr.31837.

Supplementary Tables

Supplementary Table 1. List of detected genes translocations.

Locus	Type	Genes (Exons)	Read Counts	Oncomine Variant Class	Oncomine Gene Class	Read Counts Per Million
chr12:12006495 - chr9:133729451	FUSION	*ETV6(4) - ABL1(2)*	307	Fusion	Gain-of-Function	1666.295776
chr12:12022903 - chr9:133729451	FUSION	*ETV6(5) - ABL1(2)*	19214	Fusion	Gain-of-Function	104287.3193

Supplementary Table 2. Genes and translocations tested by Oncomine Myeloid Research Assay (Thermo Fisher Scientific) and Myeloid Plus Solution (SOPHiA Genetics).

Gene	Oncomine Myeloid Research Assay (Thermo Fisher Scientific)	Myeloid Plus Solution (Sophia Genetics)
ABL1	Hot spot regions	4-9
ASXL1	full coding regions	9,11,12,14
BRAF	Hot spot regions	15
BCOR	full coding regions	/
CALR	full coding regions	9
CBL	Hot spot regions	8,9
FLT3	Hot spot regions	13-15,20
HRAS	Hot spot regions	2,3
IDH1	Hot spot regions	4
IDH2	Hot spot regions	4
KIT	Hot spot regions	2,8- 11,13,17,18
KRAS	Hot spot regions	2,3
MPL	Hot spot regions	10
NPM1	Hot spot regions	10,11
NRAS	Hot spot regions	2,3
PTPN11	Hot spot regions	3,7-13
SETBP1	Hot spot regions	4
SF3B1	Hot spot regions	10-16
SRSF2	Hot spot regions	1
TP53	full coding regions	2-11
U2AF1	Hot spot regions	2,6
WT1	Hot spot regions	6-10
CEBPA	full coding regions	full coding regions
CSF3R	Hot spot regions	full coding regions
DNMT3A	Hot spot regions	full coding regions
ETV6	full coding regions	full coding regions
EZH2	full coding regions	full coding regions
JAK2	Hot spot regions	full coding regions

RUNX1	full coding regions	full coding regions
TET2	full coding regions	full coding regions
ZRSR2	full coding regions	full coding regions
IKZF1	full coding regions	/
NF1	full coding regions	/
PHF6	full coding regions	/
PRPF8	full coding regions	/
RB1	full coding regions	/
SH2B3	full coding regions	/
STAG2	full coding regions	/
GATA2	Hot spot regions	/
MYD88	Hot spot regions	/

Table S3. Oncomine Myeloid Research Assay (Thermo Fisher Scientific) fusion content.

ABL1, ALK, BCL2, BRAF, CCND1, CREBBP, EGFR, ETV6, FGFR1, FGFR2, FUS, HMGA2, JAK2, KMT2A (MLL), MECOM, MET, MLLT10, MLLT3, MYBL1, MYH11, NTRK3, NUP214, PDGFRA, PDGFRB, RARA, RBM15, RUNX1, TCF3, TFE3.

Table S4. Myeloid Plus Solution (Sophia Genetics) fusion content (the exons involved in translocations are indicated between the round brackets).

*ATF7IP(13)-JAK2(9,11,13,15,*17,18,19), *BCR*(1,4,6,7,12,13,14,19)-*ABL1*(2,3,4), *BCR*(1,4,6,7,12,13,14,19)-*FGFR1*(11), *BCR*(1,4,6,7,12,13,14,19)-*JAK2*(9,11,13,15,17,18,19), *BCR*(1,4,6,7,12,13,14,19)-*PDGFRA*(12), *BMP2K*(14,15)-*ZNF384*(2,3,4,7), *CBFA2T3*(10,11)-*GLIS2*(4,5), *CBFB*(4,5)-*MYH11*(29,30,31,32,33,34,35), *CCDC6*(1,7)- *PDGFRB*(9,11), *CHIC2*(3)-*ETV6*(2,3), *CNTRL*(38)-*FGFR1*(11), *CREBBP*(4,5,6,7)-*ZNF384*(2,3,4,7), *CUX1*(11)-*FGFR1*(11), *DEK*(9)-*NUP214*(17,18), *EBF1*(10,13,14,15)- *JAK2*(9,11,13,15,17,18,19), *EBF1*(10,13,14,15)-*PDGFRB*(9,11), *EML1*(18)-*ABL1*(2,3,4), *EP300*(6)-*ZNF384*(2,3,4,7), *ETV6*(4,5,6,7)-*ABL1*(2,3,4), *ETV6*(4,5,6,7)-*ARNT*(3), *ETV6*(4,5,6,7)-*JAK2*(9,11,13,15,17,18,19), *ETV6*(4,5,6,7)-*NTRK3*(14), *ETV6*(4,5,6,7)-*NTRK3*(15), *ETV6*(4,5,6,7)-*PDGFRB*(9,11), *ETV6*(4,5,6,7)-*RUNX1*(1), *ETV6*(4,5,6,7)- *RUNX1*(3), *FGFR1OP*(5,6,7)-*FGFR1*(11), *FIP1L1*(12)-*PDGFRA*(12), *FOXP1*(19)-*ABL1*(2,3,4), *INPP5D*(8)-*ABL1*(2,3,4), *KAT6A*(16)-*CREBBP*(2,3), *KMT2A*(8,9,10,11)- *AFDN*(2), *KMT2A*(8,9,10,11)-*AFF1*(4,5,6,11), *KMT2A*(8,9,10,11)-*AFF3*(7,8,12), *KMT2A*(8,9,10,11)-*AFF4*(4,5,6), *KMT2A*(8,9,10,11)-*ARHGAP26*(19), *KMT2A*(8,9,10,11)- *ARHGEF12*(11,12,13), *KMT2A*(8,9,10,11)-*ARHGEF17*(2,3,4,5), *KMT2A*(8,9,10,11)-*C2CD3*(13,14,15,17), *KMT2A*(8,9,10,11)-*CBL*(10), *KMT2A*(8,9,10,11)-*CIP2A*(17), *KMT2A*(8,9,10,11)-*CREBBP*(2,3), *KMT2A*(8,9,10,11)-DCPS(2), *KMT2A*(8,9,10,11)-ELL(2,3,6), *KMT2A*(8,9,10,11)-*EPS15*(2,6), *KMT2A*(8,9,10,11)-*FOXO3*(2), *KMT2A*(8,9,10,11)- *KMT2A*(2), *KMT2A*(8,9,10,11)-*KNL1*(12), *KMT2A*(8,9,*10,11*)-*MAML2*(2,3), *KMT2A*(8,9,10,11)-*MAPRE1*(2,4,6), *KMT2A*(8,9,10,11)-*MLLT1*(2,4,5,6,7), *KMT2A*(8,9,10,11)- *MLLT10*(5,7,10,12,17), *KMT2A*(8,9,10,11)-*MLLT11*(2), *KMT2A*(8,9,10,11)-*MLLT3*(4,5,6,9,10), *KMT2A*(8,9,10,11)-*MLLT6*(8,9,12), *KMT2A*(8,9,10,11)-*NRIP3*(2), *KMT2A*(8,9,10,11)-RARA(2), *KMT2A*(8,9,10,11)-*SEP5*(2), *KMT2A*(8,9,10,11)-*SEPT6*(2), *KMT2A*(8,9,10,11)-*SEPT9*(2), *KMT2A*(8,9,10,11)-*SEPT9*(2), *KMT2A*(8,9,10,11)- *TET1*(9), *MEF2D*(7)-*CSF1R*(12), *MN1*(1)-*ETV6*(2,3), *MNX1*(1)-*ETV6*(2,3), *MYB*(8)-*GATA1*(5), *NCOR1*(35)-*LYN*(8), *NDE1*(6)-*PDGFRB*(9,11), *NPM1*(4,6)-*MLF1*(3), *NPM1*(4,6)- *RARA*(2), *NUP214*(23,26,28,29,30,31,32,34)-*ABL1*(2,3,4), *NUP98*(10,11,12,13,14)-*DDX10*(6,7), *NUP98*(10,11,12,13,14)-*HOXA9*(1,2), *NUP98*(10,11,12,13,14)-*KDM5A*(27), *NUP98*(10,11,12,13,14)-*NSD1*(6), *NUP98*(10,11,12,13,14)-*RAP1GDS1*(2,3), *NUP98*(10,11,12,13,14)-*TOP1*(8), *OFD1*(21)-*JAK2*(9,11,13,15,17,18,19), *P2RY8*(1)-*CRLF2*(1), *PAG1*(8)-*ABL2*(3,5), *PAX5*(4)-*ETV6*(2,3), *PAX5*(4)-*JAK2*(9,11,13,15,17,18,19), *PAX5*(5)-*ETV6*(2,3), *PAX5*(5)-*JAK2*(9,11,13,15,17,18,19), *PCM1*(26,36)-*JAK2*(9,11,13,15,17,18,19), *PDE4DIP*(16)-*PDGFRB*(9,11), *PICALM*(17,18,19)-*MLLT10*(5,7,10,12,17), *PML*(3,6)-*RARA*(2), *RANBP2*(18)-*ABL1*(2,3,4), *RBM15*(1)-*MKL1*(4,5), *RCSD1*(2)-*ABL1*(2,3,4), *RCSD1*(3)-*ABL1*(2,3,4), *RUNX1*(3)-*RUNX1T1*(6), *SET*(7)-*NUP214*(17,18), *SFPQ*(9)-*ABL1*(2,3,4), *SNX2*(3)-*ABL1*(2,3,4), *SPAG9*(26)-*JAK2*(9,11,13,15,17,18,19), *SPTBN1*(4)- *FLT3*(14), *SPTBN1*(4)-*PDGFRB*(9,11), *SSBP2*(5,6,8,10,16)-*CSF1R*(12), *SSBP2*(5,6,8,10,16)-*JAK2*(9,11,13,15,17,18,19), *STAT5B*(15,16)-*RARA*(2), *STIL*(1)-*TAL1*(3,4,6), *STRN*(6)-*PDGFRA*(12), *STRN3*(8,9)-*JAK2*(9,11,13,15,17,18,19), *TAF15*(6,9)-*ZNF384*(2,3,4,7), *TCF3*(11,13,15,16,17)-*HLF*(4), *TCF3*(11,13,15,16,17)-*PBX1*(3), *TERF2*(8)- *JAK2*(9,11,13,15,17,18,19), *TPM3*(8)-*PDGFRB*(9,11), *TPR*(22,39)-*FGFR1*(11), *TRIM24*(9,10,11)-*FGFR1*(11), *ZBTB16*(3,4)-*ABL1*(2,3,4), *ZBTB16*(3,4)-*RARA*(2), *ZC3HAV1*(12)- *ABL2*(3,5), *ZEB2*(9)-*PDGFRB*(9,11), *ZMIZ1*(18)-*ABL1*(2,3,4), *ZMYM2*(17)-*FGFR1*(11)
